# Predicting ‘Brainage’ in late childhood to adolescence (6-17yrs) using structural MRI, morphometric similarity, and machine learning

**DOI:** 10.1038/s41598-023-42414-5

**Published:** 2023-09-20

**Authors:** Daniel Griffiths-King, Amanda G. Wood, Jan Novak

**Affiliations:** 1 Aston Institute of Health and Neurodevelopment, College of Health and Life Sciences, Aston University, Birmingham, B4 7ET UK; 2 School of Psychology, Faculty of Health, Melbourne Burwood Campus, Deakin University, Geelong, VIC Australia; 3 Murdoch Children’s Research Institute, Melbourne, VIC Australia

**Keywords:** Psychology, Cognitive neuroscience, Intelligence, Network models

## Abstract

Brain development is regularly studied using structural MRI. Recently, studies have used a combination of statistical learning and large-scale imaging databases of healthy children to predict an individual’s age from structural MRI. This data-driven, predicted ‘Brainage’ typically differs from the subjects chronological age, with this difference a potential measure of individual difference. Few studies have leveraged higher-order or connectomic representations of structural MRI data for this Brainage approach. We leveraged morphometric similarity as a network-level approach to structural MRI to generate predictive models of age. We benchmarked these novel Brainage approaches using morphometric similarity against more typical, single feature (i.e., cortical thickness) approaches. We showed that these novel methods did not outperform cortical thickness or cortical volume measures. All models were significantly biased by age, but robust to motion confounds. The main results show that, whilst morphometric similarity mapping may be a novel way to leverage additional information from a T1-weighted structural MRI beyond individual features, in the context of a Brainage framework, morphometric similarity does not provide more accurate predictions of age. Morphometric similarity as a network-level approach to structural MRI may be poorly positioned to study individual differences in brain development in healthy participants in this way.

## Introduction

Developmental neuroscience has embraced neuroimaging studies of brain structure to characterize brain maturation and to understand how this gives rise to cognitive development. Developmental neuroimaging studies have highlighted distinct developmental trajectories of specific cortical tissues such as white matter (WM) and grey matter (GM), across different regions of the cortex^1^. The volume of cortical GM specifically shows an ‘inverted U’, nonlinear trajectory^1–4^, with pre-pubertal expansion of the cortical GM^5^ followed by a post-pubertal sustained loss of GM volume (despite synaptic density plateauing after puberty according to molecular and cellular evidence^5^). Brain maturation has specific regional trajectories; peak GM density and reductions in GM volume occur earliest in primary function areas, somatosensory and primary motor cortices, and latest in higher-order association areas, dorsolateral prefrontal cortex and superior temporal gyrus for instance^1^. Cortical thickness maturation also shows a similar trajectory, with generalized reductions over time^6–8^, in line with what would be expected from models of synaptic pruning and myelination^8^. These longstanding findings show, given these measures vary as a function of age, that an individual’s chronological age may be deduced from an MRI scan of their brain.

This is the premise of the Brainage framework, the idea that multivariate patterns of brain structure in large samples of MRI from healthy children are related to age and, by using data-driven or machine learning approaches, that association can be learnt. By applying these learnt patterns to new data, we can predict the age of an individual based on their MRI (see^9, 10^ for review). This apparent age, or more commonly termed “Brainage”, is akin to a reading age; it reflects the current observed status of the brain in terms of morphometry in comparison to ‘typical’ norms of brain structural development.

The Brainage of any individual is unlikely to be perfectly aligned to their chronological (actual) age. Differences between Brainage and chronological age may reflect normal-variation or individual differences between children. This metric of difference and/or perturbation is typically referred to as BrainageΔ (delta), the calculated difference between chronological age and apparent/predicted Brainage^9, 11^. In the case of diseased populations, this measure allows us to estimate the perturbation that the disease state has upon brain development and aging (i.e.,^11^). For instance, a BrainageΔ of zero would be indicative of an individual following a normative developmental trajectory, whilst a higher or greater BrainageΔ would represent advanced brain (and possibly cognitive) aging, a state of perturbation from the typical developmental trajectory^12^. Brain development (specifically grey matter change) follows highly ‘programmed’ trajectories^5, 13–15^ (driven in part by genetics^16–19^). Therefore, neurological disruption to the ongoing development of the brain during this period is likely to potentially symptomatic, impacting on future brain and cognitive maturation. Therefore, an approach for quantifying typical brain maturation will likely hold benefit in understanding atypical developmental patterns that hold clinical implication^20, 21^.

In recent years, data-driven estimations of the brain’s apparent age have been calculated using machine-learning approaches to detect latent patterns associated with age across several neuroimaging modalities (including structural (sMRI), diffusion (dMRI) and functional MRI (fMRI)). Utilising machine learning approaches in this way, can consider the multivariate complexities of the neurodevelopmental trajectories of these meso-scale measures. However, using regional-level data as independent features to predict age may ignore potentially relevant, higher-order multicollinearities between regions. Connectomic approaches^22^, that consider the brains’ network-level organisation, may therefore hold greater potential in predicting Brainage. Typically, connectomic approaches would utilise diffusion and functional MRI data, but these can suffer from quality issues associated with EPI sequences^23^, and also have long acquisition times and may therefore be less tolerable in clinical populations and paediatrics^24^.

This study proposes connectomic approaches to sMRI data as a potential method to use in the Brainage framework. Previous approaches utilising individual morphometric measurements from sMRI in Brainage prediction in paediatrics^20, 25, 26^ have achieved comparable prediction accuracies to those multimodal studies incorporating additional modalities with sMRI^27, 28^ (although, methodological differences preclude meaningful direct comparison across these paediatric studies). However, relatively few studies leverage connectomics-level analyses of sMRI data for Brainage prediction. Corps and Rekik^23^ utilised morphometric data (curvature, cortical thickness, sulcal depth) to produce multi-view morphological brain networks. Briefly, they investigated multiple feature-networks where ‘connections’ are the dissimilarity (absolute difference) between regions in terms of absolute feature values. This produced multiple feature networks as predictive variables. No previous studies have leveraged network-level approaches to sMRI to generate single morphometric networks as variables in predictive models of Brainage. Given the abundance of available sMRI data (T1w MRI is more readily acquired across clinical and research contexts compared to other MRI modalities and therefore may find greater translatability and application), and the effective Brainage prediction previously achieved using this data^27, 28^, there is strong rationale in trying to leverage additional predictive information from this modality of MRI, using a connectomic approach.

In the current paper, we employed a morphometric similarity mapping approach^29^ to combine multiple features into a single network, capturing higher-order morphometric organisation across the cortex. Previously these networks have been shown to be sensitive to neurodevelopmental abnormalities^30^.

Specifically, this paper generated normative Brainage models using connectomic approaches to sMRI, as outlined in King and Wood^24^, leveraging both network-level approaches whilst restricting necessary MRI sequences to a T1w sMRI. This approach may better account for absolute dissimilarity due to scaling (as in Corps and Rekik^23^) and instead capture those relationships that are indicative of coordinated cortical development and maturation^29^.

The current study evaluates the use of T1w morphometric similarity mapping, as a novel approach in predicting Brainage in a cohort of typically developing children. This study investigates whether Brainage prediction methods are more accurate when using morphometric similarity measures of the developing cortex, compared to individual morphometric measures. No previous study has benchmarked these novel Brainage approaches (using morphometric similarity) against more typical, single morphometric feature approaches.

## Results

### Dataset

This study employed data from healthy controls from the open-access Autism Brain Imaging Data Exchange cohort (ABIDE, Di Martino, Yan^31^) data from the Pre-processed Connectome Project (PCP, Bellec, Yan^32^, for full details see Pre-processed Connectome Project website http://preprocessed-connectomes-project.org/). Healthy controls were included that were < 17 years old and met strict quality control criteria (outlined below). After applying these criteria, the remaining cases from the ABIDE dataset used in the current analyses consisted of 327 healthy controls, with a mean age of 12.4 ± 2.5 years. (see Table 1). We utilized the Freesurfer^33^ processed outputs supplied by the PCP. This provides cortical morphometry measures across regions of the Desikan-Killiany atlas^34^.

**Table 1:** Demographic information from entire cohort (n = 327 from ABIDE dataset) and the internal training and validation cohorts and the independent test cohort.

	Entire cohort	Internal training cohort	Internal validation cohort	Test cohort
n	327	204	41	82
Mean age (yrs. ± SD)	12.4 ± 2.5	12.7 ± 2.5	12.3 ± 2.6	11.8 ± 2.4
Min. age (yrs.)	6.5	6.5	7.3	7.3
Max. age (yrs.)	16.9	16.9	16.9	16.6
Sex (M:F)	259:68	164:40	31:10	64:18
Mean IQ (IQ ± SD)	110 ± 15^a^	110 ± 13^b^	111 ± 11^c^	109 ± 14^d^

^a^Available for n = 308, ^b^available for n = 192, ^c^available for n = 40, ^d^available for n = 76.

### Model evaluation

**Figure 1:**
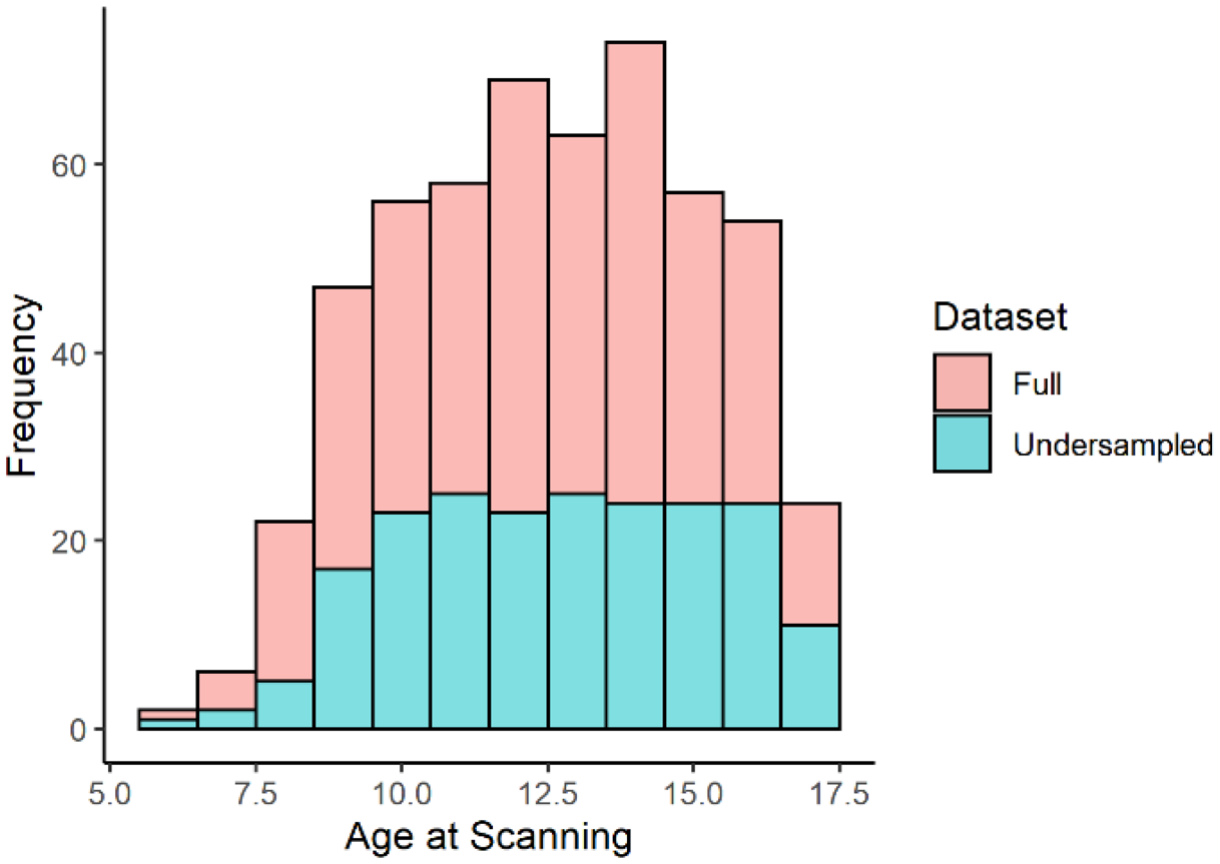
Age of participants in the entire cohort versus those in the training cohort. This graph highlights the under sampling of the training set based on age. It was important to ensure that the training cohort did not disproportionately represent any one specific age group, just because of the greater frequency of that age group in the full dataset. A ‘flatter’ distribution of age was selected in the training set by under sampling age bands that are ‘overrepresented’ in the overall dataset. This was less successful at the extreme ‘tails’ of childhood (approximately less than 8 years and greater than 16.5 years) where less data was available to sample from. N.B for visualization purposes, this graph is re-binned to bins of width 1 year.

To evaluate the Brainage models derived from different morphometric feature sets, specifically morphometric similarity, compared to individual morphometric features, the ABIDE cohort were divided into training and independent test cohorts in the ratio of 3:1 (n = 245 & n = 82 respectively). The training cohort was further subdivided into an internal training and validation cohort at a ratio of 5:1 (n = 204 & n = 41). Selection of the training-set was pseudo-random to enable under sampling based on age (see Fig. 1).

Prediction utilized 10 different feature-sets (See Table 2); i–vii) each of the individual morphometric features, viii) all features, ix) nodal-level strength of morphometric similarity and x) edge-level weights of the morphometric similarity, with each model having chronological age at scanning as the dependent variable. Across models, performance was evaluated based upon reducing mean absolute error (MAE) and maximizing predictive R^2^.

**Table 2:** Features sets used to produce Brainage models.

Feature set	n features
Individual morphometric features	
1. Surface area	68^a^
2. Curvature index	68
3. Folding index	68
4. Gaussian curvature	68
5. Mean curvature	68
6. Cortical thickness	68
7. Cortical volume	68
8. All individual features	476^b^
Morphometric similarity	
9. Morphometric similarity nodal strength	68
10. Morphometric similarity edge weight	2278^c^

N.B. ^a^ Number of regions in the Desikan Killiany atlas, ^b^ Number of regions time number of individual morphometric features, ^c^ Number of off-diagonal elements of the connectivity matrix.

### ML algorithm and kernel selection

**Figure 2:**
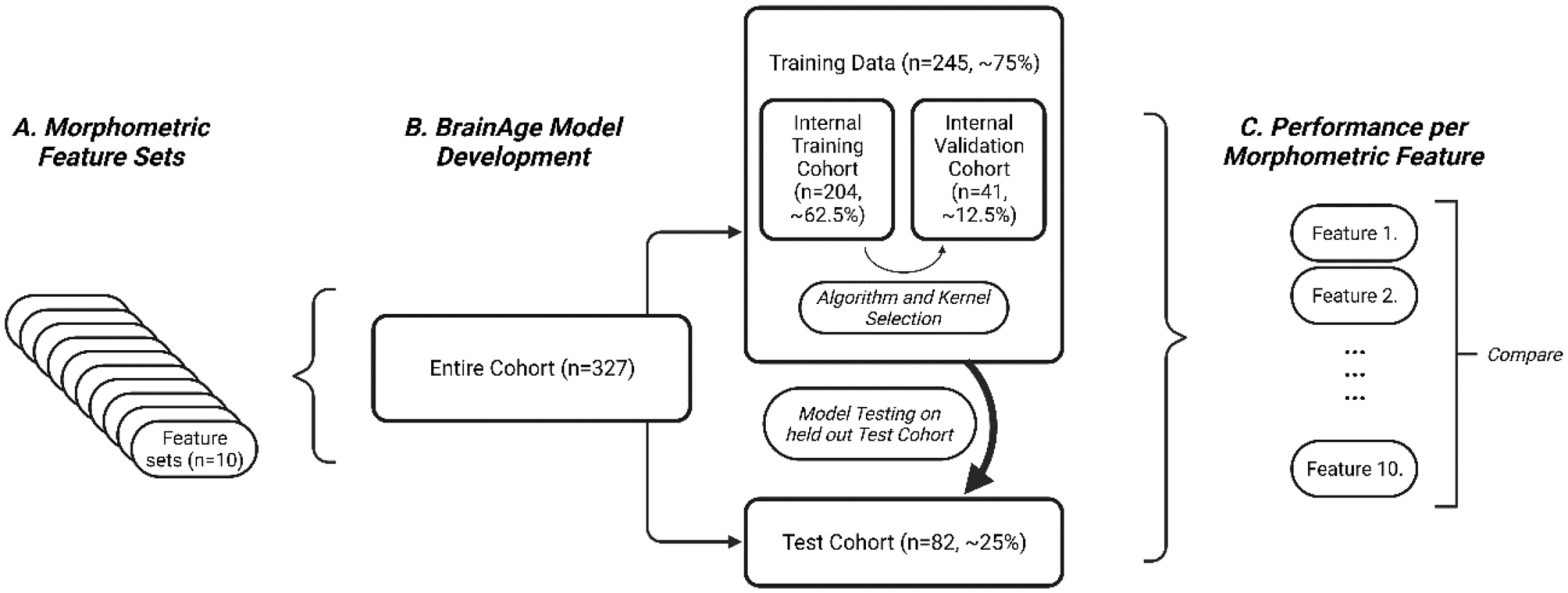
Flowchart detailing the use of data in the current study for internal validation, training, and testing. Figure created with BioRender.com.

Brainage prediction was conducted across two, kernel-based regression approaches; a) Gaussian Processes Regression (GPR) and b) Relevance Vector Regression (RVR).These were selected as both are commonly used in the literature^9, 10, 35^, and non-linear and/or kernel-based algorithms typically outperform linear approaches (likely due to the multicollinearity in morphometric measures^36, 37^. Two different kernels were tested for each algorithm: a) laplacedot (Laplace radial basis kernel) and b) RBFDot (Gaussian radial basis function). Internal training and validation were conducted to select the machine learning algorithm and kernel, based upon performance when trained on the internal training cohort and evaluated on the internal validation set. The hold out test cohort was not included in this process. Figure 2 details this further.

Table 3 highlights the performance of each model in both the internal training and validation sets. For all feature sets, Gaussian processes regression (paired with either the laplacedot or RBFdot) seemed to perform best on the validation set. The model (algorithm + kernel) which performed best on the internal validation set for each feature set was evaluated on the independent test cohort to estimate performance for each feature set.

**Table 3:** Performance of Brainage models trained on different feature sets and assessed on Internal Training and Validation cohorts. Performance on internal validation cohort informed the selection of ML algorithm and kernel.

Feature	Algorithm	Kernel	Internal training (n = 204)		Internal validation (n = 41)		Feature	Algorithm	Kernel	Internal training (n = 204)		Internal validation (n = 41)	
			MAE (yrs.)	Pred. R^2^	MAE (yrs.)	Pred. R^2^				MAE (yrs.)	Pred. R^2^	MAE (yrs.)	Pred. R^2^
Surface area	GuassPrc	laplacedot	**1.40**	**0.55**	**1.96**	**0.06**	Cortical thickness	GuassPrc	laplacedot	**1.07**	**0.72**	**1.44**	**0.53**
		rbfdot	1.40	0.55	1.96	0.06			rbfdot	1.07	0.72	1.44	0.53
	RVM	laplacedot	1.51	0.45	2.28	− 0.19		RVM	laplacedot	1.24	0.64	1.96	0.09
		rbfdot	1.47	0.48	2.23	− 0.17			rbfdot	1.20	0.65	1.95	0.10
Curvature index	GuassPrc	laplacedot	**1.40**	**0.57**	**2.06**	**0.02**	Cortical volume	GuassPrc	laplacedot	**1.23**	**0.64**	**1.56**	**0.37**
		rbfdot	1.41	0.56	2.06	0.02			rbfdot	1.24	0.63	1.56	0.37
	RVM	laplacedot	1.41	0.49	2.54	− 1.20		RVM	laplacedot	1.42	0.52	1.68	0.27
		rbfdot	1.28	0.57	2.59	− 1.22			rbfdot	1.44	0.50	1.66	0.28
Folding index	GuassPrc	laplacedot	**1.32**	**0.62**	**1.97**	**0.09**	All individual features	GuassPrc	laplacedot	1.05	0.74	1.37	0.49
		rbfdot	1.29	0.63	1.97	0.09			rbfdot	**1.06**	**0.73**	**1.37**	**0.49**
	RVM	laplacedot	1.32	0.52	3.22	− 2.05		RVM	laplacedot	1.26	0.60	1.43	0.51
		rbfdot	1.39	0.48	3.02	− 1.72			rbfdot	1.24	0.62	1.40	0.53
Gaussian curvature	GuassPrc	laplacedot	**1.36**	**0.60**	**1.82**	**0.23**	Morphometric similarity: nodal strength	GuassPrc	laplacedot	1.40	0.56	1.77	0.31
		rbfdot	1.34	0.61	1.82	0.23			**rbfdot**	**1.39**	**0.56**	**1.77**	**0.31**
	RVM	laplacedot	1.23	0.59	3.85	− 3.41		RVM	laplacedot	1.32	0.57	2.01	0.05
		rbfdot	1.25	0.58	3.53	− 3.01			rbfdot	1.18	0.64	2.03	0.03
Mean curvature	GuassPrc	laplacedot	**1.40**	**0.56**	**1.91**	**0.19**	Morphometric similarity: edge weights	GuassPrc	laplacedot	1.20	0.69	1.73	0.32
		rbfdot	1.42	0.55	1.91	0.19			**rbfdot**	**1.20**	**0.68**	**1.73**	**0.32**
	RVM	laplacedot	1.44	0.49	2.20	− 0.10		RVM	laplacedot	0.36	0.96	1.82	0.31
		rbfdot	1.62	0.37	2.14	− 0.04			rbfdot	0.33	0.97	1.82	0.31

Pred. R^2^, Predicted R^2^; GuassPrc, Gaussian processes regression; RVM, Relevance Vector Machine; laplacedot, Laplace radial basis kerne; rbfdot , Gaussian radial basis function. Negative Pred. R^2^ values (in red) represent where performance was poorer than prediction using only the mean. Bold indicates for each feature set the combination of algorithm and kernel which produced the most favourable results in the validation set (based on predicted R^2^ as the evaluation metric).

### Model evaluation on independent test cohort

**Figure 3:**
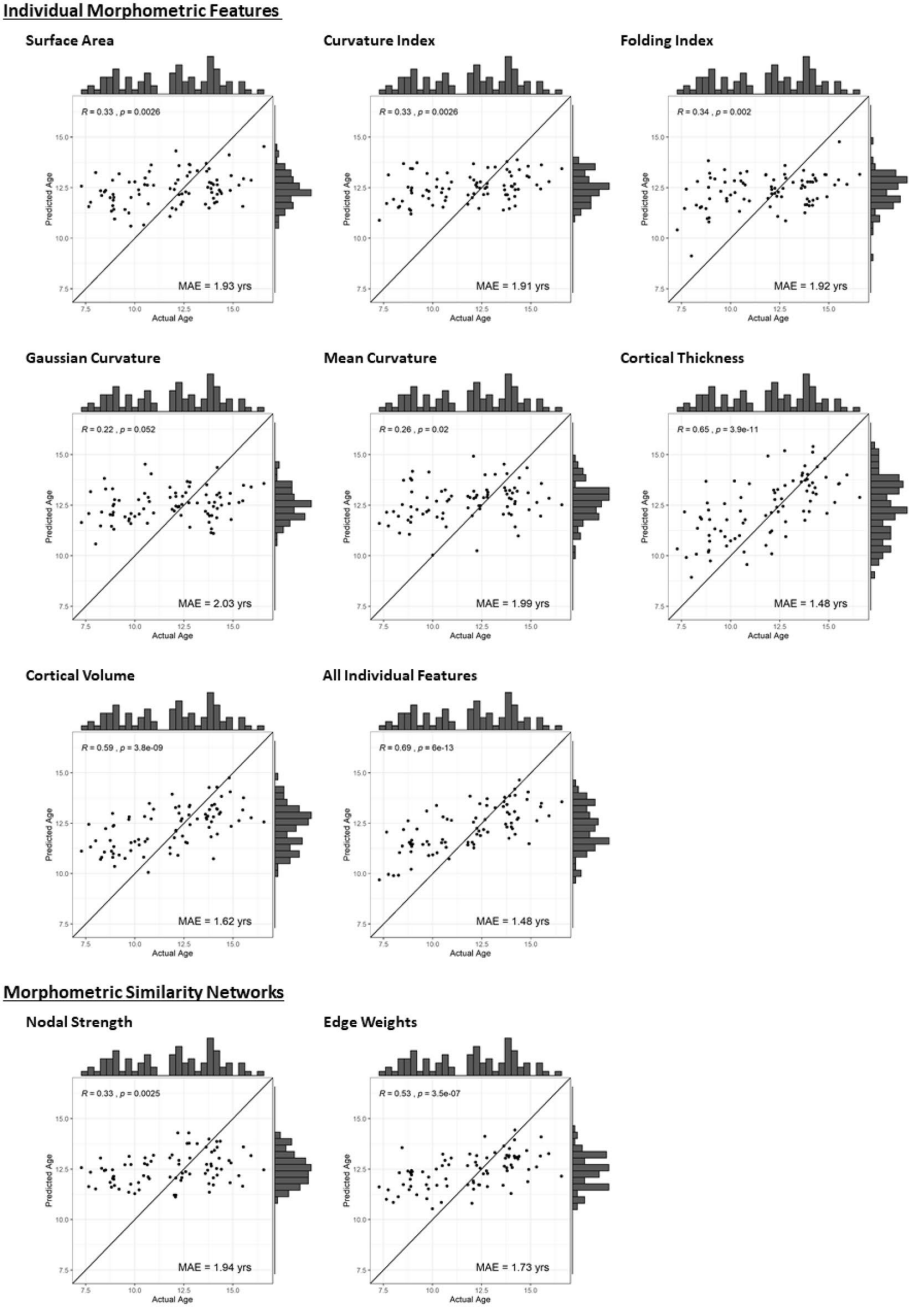
Performance of Brainage prediction on independent testing cohort, for each of the feature sets, including (**a**) individual morphometric features and (**b**) network features based on Morphometric Similarity. Chronological age is plotted against the age predicted by the model. Plotted line is where actual age = predicted age (x = y), which would represent perfect prediction.

Models were trained on the training cohort (n = 245) and then evaluated on the independent test cohort (n = 82). Table 4 highlights the results of this model testing, with data plotted in Fig. 3. Evaluations suggest that Gaussian and mean curvature performed poorest, with prediction worse than a model of just the mean (R^2^ = − 0.05 & − 0.09 respectively). Morphometric Similarity edge weights, cortical volume, thickness and all individual features performed strongest (R^2^ = 0.19, 0.29, 0.37 & 0.39 respectively). Based on random resampling of the data (training/testing cohorts), we calculated mean predicted R^2^ of models and 95% confidence intervals (CI) of these values. Only models based upon Morphometric Similarity edge weights, cortical volume, thickness and all individual features had 95% CI that did not cross predictive R^2^ = 0. Performance across the resampling for these models was variable, as can be seen in the 95% CI.

**Table 4:** Performance of Brainage models trained on different feature sets and assessed on Training and Test samples.

Feature	Training cohort (n = 245)		Test cohort (n = 82)				
	MAE (yrs.)	Pred. R^2^	MAE (yrs.)	Pred. R^2^	Mean^a^ pred. R^2^	Pred. R^2^ (95% CI^b^)	*p*-value^c^
Surface area	1.41	0.54	1.93	0.04	0.04	(− 0.06 to 0.15)	0.029
Curvature index	1.39	0.57	1.91	0.02	− 0.03	(− 0.16 to 0.08)	0.233
Folding index	1.35	0.60	1.92	0.06	0.04	(− 0.11 to 0.15)	0.025
Gaussian curvature	1.36	0.59	2.03	− 0.05	0.02	(− 0.09 to 0.13)	0.067
Mean curvature	1.38	0.57	1.99	− 0.09	0.01	(− 0.10 to 0.12)	0.076
Cortical thickness	1.07	0.72	1.48	0.37	0.41	(0.28 to 0.52)	** < 0.001**
Cortical volume	1.21	0.65	1.62	0.29	0.26	(0.13 to 0.38)	** < 0.001**
All individual features	1.03	0.74	1.48	0.39	0.38	(0.27 to 0.47)	** < 0.001**
Morphometric similarity: nodal strength	1.35	0.58	1.94	0.02	0.10	(− 0.02 to 0.23)	0.005
Morphometric similarity: edge weights	1.16	0.70	1.73	0.19	0.23	(0.12 to 0.31)	** < 0.001**

Pred. R^2^, Predicted R^2^, CI, Confidence Interval. Training sample represents the combination of both training and validation samples. Negative predicted R^2^ values (in red) represent where performance was poorer than prediction using only the mean. ^a^Mean and ^b^Confidence intervals of predictive R^2^ values are based upon 100 random partitions (training/ testing cohorts) of the data. ^c^*p*-value derived from 1000 permutations of age at scanning in the full training sample (see methods below, bold = significant at α < 0.05/10).

Null models were produced by permuting age in the training cohort and evaluating on actual testing data. The mean predictive R^2^ values of the resampled models, and the distribution of R^2^ values from the permuted ‘null’ cases allowed calculation of *p*-values, where models performed above random noise in the data. Again, only models based upon Morphometric Similarity edge weights, cortical volume, thickness and all individual features produced models which performed significantly above null models.

### Prediction using density thresholded morphometric similarity

Given that correlation-derived networks may represent both ‘real’ statistical associations and potential noisy/spurious associations^38^ we also tested prediction based upon edge-level Morphometric Similarity, thresholded at an individual-level, at multiple network densities; from top 5% edges to 50% in steps of 5%. For all densities, in terms of both predicted R^2^ and MAE, GuassPrc outperformed the RVM algorithm in internal validation procedures. Irrespective of kernel, prediction performed equally well (to 2dp) for all densities tested (All models 5–50% density: MAE = 1.73 yrs, Pred. R^2^ = 0.32) on the internal validation. Given the predictive accuracy remains constant even when the network is thresholded to enforce greater sparsity, this suggests that the top 5% of edges in terms of weight are those that are most sensitive to individual differences due to age. As the performance did not change compared to the original, unthresholded network, we used the unthresholded network for the presented analyses here, including performance evaluation on the hold out testing cohort.

### Potential biases in BrainageΔ

**Figure 4:**
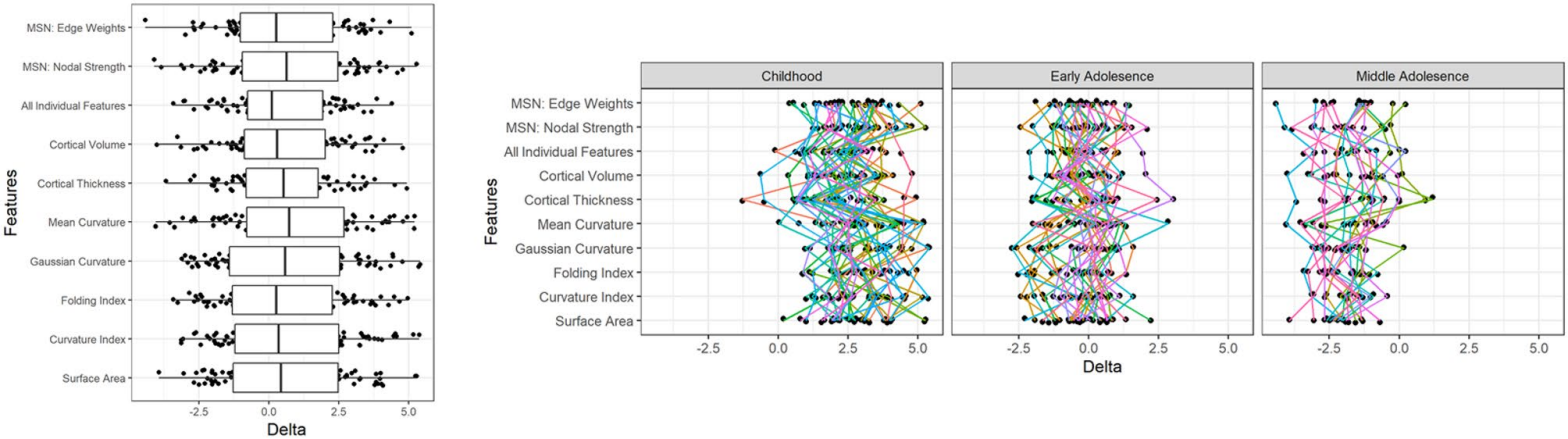
Plots showing BrainageΔ for the testing cohort; across each model based on different feature sets (left) and when divided into developmental periods of childhood (5–11 years), early adolescence (11–14 yrs) and middle adolescence (14–17 years).

BrainageΔ was calculated as the absolute difference between chronological (actual) actual and predicted Brainage, in the testing cohort. This indexes the degree to which an individual diverges from age-expected, brain development (combined with model error). As expected, due to the ‘healthy’ nature of the participants, many of these values were close to zero, although there was large variability (across all feature sets; mean (SD) = 0.62(2.11), median = 0.44). Whilst the variation in BrainageΔ at the group-level was similar across models, Fig. 4 also shows that, at an individual participant level there was large variability in BrainageΔ between models. That is to say, a participant with a high BrainageΔ for cortical thickness, could have a smaller or close-to-zero BrainageΔ for another morphometric measure. Visual inspection of performance on test data highlighted that across many of the feature sets there was a flatter gradient in the data (actual vs predicted age) compared to the line of x = y (perfect prediction) suggesting an overestimation of age in younger children and an under estimation of age in the older adolescent participants. This was further seen in Fig. 4 when individual BrainageΔ profiles (across models) when divided by age group (childhood, early adolescence, middle adolescence). Given age-related bias in previous studies^12, 39, 40^, we controlled for age in the remaining analyses of BrainageΔ using partial correlations, a correction approach which has been identified to be as effective as correcting the predicted values or correcting the model for these biases^41^.

(A) Potential Biases in BrainageΔ: Sex.

Potential sex differences in BrainageΔ estimation were investigated using linear models controlling for actual age. Across all models, the effect of sex did not meet significance.

(B) Potential Biases in BrainageΔ: Motion.

To evaluate potential bias in the models from motion we used the Entropy Focus Criterion (EFC^42^) as a proxy for motion derivable from T1w images. EFC uses the Shannon entropy of voxel intensities to typically quantify the amount of motion present^43^, specifically through the sensitivity to motion-induced artifacts (e.g., ghosting and blurring induced by head motion). MRI autofocusing techniques based on EFC optimisation have been shown to reduce motion artifacts effectively^42^.

Average correlation between BrainageΔ and EFC across the models was close to zero ($\overline{r }$ = − 0.03), with no correlation reaching statistical significance (Table 5).

**Table 5:** Statistical associations between BrainageΔ and covariates for each feature set.

Covariate	n	Feature set	Pearson		Spearman Rho	
			R	*p*	r	*p*
Motion^a^	82	Surface area	− 0.05	0.65	− 0.16	0.16
		Curvature index	− 0.04	0.74	0.01	0.93
		Folding index	0.06	0.57	0.04	0.71
		Gaussian curvature	0.03	0.76	0.05	0.67
		Mean curvature	0.01	0.94	0.04	0.75
		Cortical thickness	− 0.06	0.62	− 0.08	0.48
		Cortical volume	− 0.23	0.04	− 0.06	0.61
		All individual features	− 0.07	0.51	− 0.11	0.31
		Morphometric similarity: nodal strength^b^	0.04	0.73	− 0.08	0.47
		morphometric similarity: edge weights^b^	− 0.03	0.77	− 0.06	0.61
IQ^a^	56	Surface area	0.23	0.08	0.24	0.08
		Curvature index	0.10	0.47	0.09	0.50
		Folding index	− 0.05	0.72	− 0.06	0.66
		Gaussian curvature	0.03	0.83	− 0.07	0.60
		Mean curvature	− 0.04	0.76	0.02	0.89
		Cortical thickness	− 0.04	0.77	0.00	0.99
		Cortical volume	0.00	0.99	0.08	0.57
		All Individual features	− 0.14	0.30	− 0.17	0.23
		Morphometric similarity: nodal strength^b^	0.05	0.69	0.07	0.62
		Morphometric similarity: edge weights^b^	− 0.06	0.65	0.05	0.71

Covariate	n	Feature set	Statistic	*P*		
Sex^a^	82	Surface area	− 1.46	0.15		
		Curvature index	1.53	0.13		
		Folding index	0.33	0.74		
		Gaussian curvature	0.14	0.89		
		Mean curvature	1.25	0.22		
		Cortical thickness	− 0.56	0.57		
		Cortical volume	1.64	0.10		
		All individual features	− 0.16	0.88		
		Morphometric similarity: nodal strength^b^	− 0.39	0.70		
		Morphometric similarity: edge weights^b^	− 0.32	0.75		

^a^Controlling for actual age, ^b^ derived from unthresholded Morphometric Similarity, Bold indicates those tests significant at Bonferroni corrected α-level = .000833.

### Exploratory relationship with cognition

To evaluate BrainageΔ as a putative measure of meaningful variation due to individual differences, we investigated relationships between BrainageΔ and individual IQ using partial correlations (using actual age as a confound to address age-bias in the BrainageΔ measure). A limited number (n = 56) of children in the test sample had valid measures of IQ. Neither morphometric similarity derived BrainageΔ or that calculated from models of individual features correlated with IQ. Whilst still non-significant, the greatest effect was found with surface area.

### Combining models for Brainage prediction

Exploratory analyses were conducted to combine the feature sets from the best performing Brainage models to investigate whether models provided incremental increase in Brainage prediction by predicting unique variance in age. Combining cortical thickness, cortical volume and morphometric similarity edge weights, as the best performing individual features using Gaussian processes regression (with rbfdot kernel), training on the training cohort resulted in comparable performance to the best performing models seen in Table 4 (MAE = 1.06 yrs., Pred R^2^ = 0.74). On the independent testing cohort, performance dropped significantly (MAE = 1.59 yrs, Pred. R^2^ = 0.31), performing better than the Cortical volume and morphometric similarity weight models but still outperformed by the cortical thickness model. The BrainageΔ estimates from this model (thickness + volume + morphometric similarity edge weights) were still biased by age (Pearson’s *r* = − 0.93, *p* =  < 0.00001), with no discernible relationship to individual differences in cognition (Pearson’s *r* = − 0.17, *p* = 0.23).

## Discussion

To our knowledge, this is the first study to construct Brainage models derived from network-level descriptions of neuroanatomical organization across the cortex. These models using morphometric similarity as a basis for predicting chronological age did not outperform non-network models, using ‘standard’ morphometric features.

Specifically, the morphometric similarity model was outperformed (in terms of lowest MAE and highest predicted R^2^) by models which included all individual structural features, followed by cortical thickness and volumetric models. The morphometric similarity edge weight model did, however, perform significantly better than null models on testing data, suggesting that these Brainage models are capturing ‘real’ patterns of variation indicative of age. The fact it was outperformed by a model where all individual features were entered separately indicated that the morphometric similarity model does not necessarily capture additional variation. One could argue that the morphometric similarity nodal-level model represents a more efficient model compared to the all-features model (given the smaller size of the feature set)—but given the 68-feature cortical thickness model also outperformed the morphometric similarity model, it would not be a more effective data reduction approach either.

The best performing (individual) structural feature for age prediction in this study was cortical thickness. Conversely, in a previous report of lifespan (8–96 yrs) Brainage prediction, in the 8–18 yr old group, across all approaches using either cortical area, thickness or volume, the greatest performance (i.e. lowest mean prediction error) was actually seen using brain volume model^44^. However, across the six prediction techniques investigated in^44^, cortical thickness models outperformed cortical volume models in 3/6 methods. This similar performance is maybe unsurprising given that cortical thickness and surface area both independently contribute to volume measurements^45^ and that volume measurements can be estimated from the product of cortical thickness and surface area measurements at all locations across the cortical mantle (in surface-based approaches)^46^. The findings of this analysis, alongside previous reports^20, 21, 25^, highlight the sensitivity and importance of cortical thickness in late childhood to adolescent development compared to surface area and curvature-based cortical measures or even novel methods of morphometric similarity.

All other tested structural features (Surface Area, Curvature Index, Folding Index, Gaussian Curvature, Mean Curvature) did not significantly outperform null models. This suggests that these measures are less sensitive to developmental changes within the window of late childhood to adolescence (6–17 yrs). For example, surface area and gyrification index measures may be more relevant to the developmental changes found from the third trimester to the early post-natal period as evidenced in imaging of term and preterm infants^47^. Certainly gross (rather than ROI) structural measures highlight that differing rates of change (and peak velocity of change) vary across developmental periods, with different measures being potentially more discriminatory over these periods^48^. Therefore, current results would highlight that these additional features are less important during this developmental window.

We also found that combining best performing models (cortical thickness, volume, and morphometric similarity edge weights) resulted in a drop in performance compared to the cortical thickness model. Whilst not a direct statistical comparison, this suggests that these models do not capture independent variance in relation to age. This seems to disagree with previous work^20^ which found that joint covariation across multiple structural features predicted variance in age independently from variance in individual features. It is unclear to the degree that the joint and distinct variation features used in^20^ are directly comparable to representations learnt by the machine learning techniques in this study, or even how they relate to the morphometric similarity approach outlined here and in^29^. Comparison of these different approaches to data ‘fusion’ across morphometric measures will be required to reconcile the potential differences between these study findings.

As well as feature sets affecting Brainage estimation, the machine learning or prediction workflow is also a key factor. This study found GPR to outperform the RVR approach. These methods were selected as they have been shown to outperform other linear approaches^36^, including in paediatrics^37^. On the surface, our finding seems to contradict other, comparative analyses of machine learning models in predicting Brainage using morphometric data who found RVR to systematically outperform GPR^49^. However, the one scenario in which GPR did outperform RVR in^49^, was in the test case with the smallest number of participants, closer to that of the sample size used here. Therefore, machine learning model will be an important consideration for future use cases.

Currently, only two other study predicted Brainage from sMRI in the ABIDE cohort^23, 50^. Using a complex network approach to T1w MRI, in 7–20 yr olds, Bellantuono et al.^50^ achieved a MAE of 1.53 years using deep learning models. The slightly larger age range means that the MAE are not entirely comparable with the current study, although the present study has outperformed this. It is important to note that the network approach to T1w MRI in this study modelled correlation grey-levels of the image rather than structural metrics.

When BrainageΔ was calculated for the test cohort, there was great variability in of an individual’s delta values for each of the feature sets; there appeared to be little consistency in these values between models. The varying individual profiles of BrainageΔ has two possible explanations. Firstly, BrainageΔ represents the combined measure of individual variance from the expected developmental trajectory plus the error in the normative age model. It therefore may be the case that the random error in each of the models is resulting in variance in BrainageΔ, across feature sets, at the individual level. This could have potential implications for the comparison of studies utilizing the Brainage measure if there is limited consistency in these measures within an individual participant. Alternatively, a potentially more interesting explanation, is that each Brainage model is indexing relevant divergences/individual differences in different aspects of cortical architecture, resulting in between model variance in BrainageΔ. This could prove to be useful in neurological conditions that influence difference aspects of brain development/organization in the paediatric brain, for instance a Brainage model based upon MRI measure of white matter may be more sensitive to differences from normative brain development in acute demyelinating disorders such as multiple sclerosis. In this scenario, multiple BrainageΔ’s from different features, or even imaging modalities could be used, as potential biomarkers of clinically relevant outcomes.

However, it is difficult to statistically test each of these explanations (model error vs meaningfully different divergences) because there are a limited number of models used in any one study. Future meta-analytic research could compare within-participant BrainageΔ values across feature sets, whilst controlling for the MAE of the model themselves, in order to isolate ‘real’ within-individual variation in the BrainageΔ measure. Future studies could also use multiple (even multi-modal) Brainage models and use the feature specific BrainageΔ’s as individual predictors in regression models, to assess unique predictive variance offered by each feature.

A strength of the current study was the extensive assessments of the morphometric similarity model, in the context of the Brainage framework across multiple analyses;

- we tested against individual structural features, in a held-out testing cohort,
- we assessed robustness of performance in terms of sampling (assessing the 95% CI of performance) and against meaningful null models and,
- we investigated correlations between BrainageΔ and biases/cognition in the independent testing sample.

As noted by^50^, ABIDE is also a particularly challenging dataset for the estimation of Brainage, due to the number of different sites and acquisition protocols. For future Brainage studies of development, this high bar should at least be maintained, with future improvements seen by validating on an entirely independent dataset (for example as seen in^20^).

An outstanding question for future research is whether there is need for models such as morphometric similarity as the popularity for deep learning/machine learning approaches become more prevalent. Fisch et al.^51^ report the results of the Predictive Analytic Competition (2019) for predicting chronological age from structural neuroimaging. They highlight the high-performing nature of neural networks for deep machine learning within the Brainage framework. Morphometric Similarity models the covariance structure of anatomical MRI features in a way which is constrained by anatomy (either using ROIs or voxels for instance) typically using a very specific, linear approach to these covariances/similarity (Pearson’s correlation coefficients). The morphometric similarity model has been shown to capture biologically meaningful information^29^ however, imposing such a model as an anatomical-prior may be redundant in analysing larger sample sizes with machine learning approaches. The machine learning/deep learning approaches that are becoming more popular in the neuroimaging literature, when fed all the individual features which are used to construct the morphometric similarity network (as we have done here), should be able to recover any covariance between structural features (even beyond linear relationships) that is captured by the morphometric similarity network approach. This may be supported by the results reported here, with greater performance seen for a model using all features compared to the morphometric similarity models.

One way in which studies have tried to identify the functional relevance of BrainageΔ is through associations with outcomes such as cognition. However, we found no relationship between Brainage as a measure of individual-difference and cognition in this typically developing cohort. Interestingly, more accurate Brainage models did not hold any greater associations with cognition. These results suggest that, when these models are generalized to ‘novel’ cases (in this situation the testing cohort), the resultant BrainageΔ measures do not hold information pertinent to individual differences in cognition. Ball et al.^52^ also reported no significant relationship between individual-level BrainageΔ (derived from voxel-based cortical thickness, volume and surface area) and cognitive abilities (as measured by the NIH Toolbox Cognition Battery). They hypothesized that this may be due to the methods they utilised which maximized the captured age-related variance in neuroanatomical measures, and that cognition-related variance (non-age related) may be captured by a different, orthogonal pattern of neuroanatomical correlates. However other studies have also found no convincing relationship between Brainage and cognition in typical developing children^25, 53^. Of those that did find a relationship in developing cohorts^54, 55^, these associations were small to moderate in size and thus likely require large sample sizes to reliably detect^53^. Finding neurodevelopmental outcomes for which this approach offers meaningful insight is key in providing functional utility of this approach as a relevant biomarker, but it seems that cognition is an unlikely candidate for this. It is perhaps unsurprising though, given that these models have been optimized for more accurate predictions of age, rather than optimized to capture phenotypes of interest^56^. A more appropriate approach for future studies, may be to establish models that directly capture variance in cognitive ability, rather than capturing an indirect biomarker^52, 56^.

Whilst the current study investigated morphometric similarity in the Brainage framework as an indirect marker of cognition, the current finding also adds to an increasing literature which questions the direct association between the meso-scale organization of morphometry across the cortex (as captured by morphometric similarity) and measures of cognitive ability. Outside of the Brainage framework, we also found no relationship between these measures and cognitive abilities^24^, failing to replicate the findings of^29^. However, a recent study of adolescence has highlighted the predictive validity of morphometric similarity across cognition/intelligence and psychiatric symptoms^57^, and so this is still very much an open area of research.

## Conclusion

Overall, whilst these network models of sMRI, using morphometric similarity, seem to mature as a function of age in typical neurodevelopment^29^, and capture meaningful variation indicative of chronological age in the Brainage framework, these networks are not most sensitive to the changes across childhood compared to other, more simplistic features, for instance cortical thickness measures.

## Methods

### Materials and data availability

The data used in this research was acquired through the public Autism Brain Imaging Data Exchange (ABIDE, Di Martino, Yan^31^) database. Specifically, we used the ABIDE data release as shared by the Preprocessed Connectome Project (PCP, Bellec, Yan^32^. For full details and access see Pre-processed Connectome Project website http://preprocessed-connectomes-project.org/). Results and metadata of the current study are available on request from Dr Griffiths-King. The R code is also available from the authors upon request, however all open-source packages used in the study are listed here: *data.table, scales, psych, ggplot2, neuroCombat, ggseg, dplyr, ggpubr, ggExtra, kernlab, ppcor, PupillometryR, tidyr*.

### Ethics statement

The database has de-identified all the patient health information associated with the data. A favourable ethical opinion was granted by Aston University Research Ethics Committee (UREC) for the secondary analysis of the ABIDE datasets (no. 1309).

### Participants

The ABIDE dataset consists of a large sample of 532 individuals with autism spectrum disorders and 573 typical controls, composed of MRI (functional and structural) and phenotypic information for each subject, accumulated across 17 independent neuroimaging sites. The scan procedures and parameters are described in more detail on the ABIDE website (http://fcon-1000.projects.nitrc.org/indi/abide/). We applied four inclusion criteria to this dataset, only including subjects who; a) passed a strict MRI quality control of raw structural MRI (see below), b) were recorded as controls within the ABIDE database, c) at time of scan were aged < 17 years old and d) had pre-processed Freesurfer data available as part of the PCP data release. This resulted in a total cohort of n = 327. Group demographics can be seen in Table 1. The ABIDE cohort had a mean IQ of approximately 110, as measured across multiple age-appropriate IQ tests (See ABIDE documentation for details).

### Data quality check

The PCP data release includes image quality metrics (IQMs) which provide quantitative ratings of the quality of the raw T1-weighted (T1w) MR images. These are calculated using the Quality Assessment Protocol software (QAP, Shehzad, Giavasis^58^). The ABIDE dataset includes data from 17 recruitment sites, and such there is potential for ‘batch effects’ on QA metrics^43^. We used the six spatial anatomical QA measures. Hence, all QA metrics were centred (mean subtracted) and scaled (divided by standard deviation) within sites, then recoded to increased values representing greater quality. This results in metrics which can be compared between sites. For each subject, QA metrics were coded as failed if they had a Z score below − 1.5 (indicating quality which was 1.5SD below the mean). We included subjects if they had zero or one QA metric that fell below this quality metric. Of the ABIDE cases who were recorded as a) controls and b) being younger than 17 years of age at scanning (n = 361), 14 subjects were removed due to having greater than one QA metric fall below the 1.5SD cut off (20 participants also had no Freesurfer data available, resulting in the final ABIDE dataset of n = 327). Further details of the automated QA measures which are included can be found here: http://preprocessed-connectomes-project.org/abide/quality_assessment.html and http://preprocessed-connectomes-project.org/quality-assessment-protocol.

### Structural MRI processing with freesurfer

3D tissue segmentation and estimation of morphometric features from T1w MR images was conducted using an established pipeline (Freesurfer version 5.1; details are published elsewhere Fischl, van der Kouwe^59^, see Fischl^33^ for review). Briefly, T1w images were stripped of non-brain tissues^60^, GM/WM boundaries were tessellated and topology was automatically corrected^61, 62^. Finally, deformation of this surface was performed, to optimally define the pial (Cerebro-spinal fluid/GM) and white (GM/WM) surfaces using maximum shifts in intensity gradients to define boundaries of these tissue classes^63–65^. Morphometric measures were derived from these surface-based models using standard methods from the Freesurfer recon-all pipeline^33^.

### Data harmonization

Multi-site imaging data harmonization was conducted using the neuroComBat package^66, 67^, an R implementation of the ComBat method^68^ for removing batch-effects (i.e. site-effects) in neuroimaging data. This was applied to the participant by ROI matrix for each morphometric feature individually, to remove site effects found in the ABIDE data, whilst protecting biological variation due to age. Fortin and colleagues have shown this approach to be effective in removing site effects in multi-site imaging data even when the biological covariate of interest (in this case age) is not balanced across sites^66, 67^. These site-corrected morphometric measures were used for a) estimation of morphometric similarity networks and b) for the individual feature models.

### Estimating morphometric similarity

Previously morphometric similarity was estimated from morphometric features measured in-vivo by both structural and diffusion MRI^29^. However, we highlighted significant correspondence between this multimodal morphometric similarity and that estimated with only features obtainable from a T1w MRI^24^ and recent papers have similarly adopted this T1w-only approach^69^, as does the current study.

To estimate morphometric similarity, the nodes for network construction were the ROIs from the Desikan-Killiany atlas^34^. At an individual-level, the seven morphometric features estimated for each node can be expressed as a set of n vectors of length 7, with each vector as a different anatomical region (n = 68), and each element of the vector a different morphometric measure. To normalize measures within this length 7 vector, each morphometric feature is demeaned, and SD scaled across the 68 regions, using Z-scores. A correlation matrix was generated for each participant, where each element of the matrix is the correlation between the feature vectors for every possible pairwise combination of regions. This correlation matrix represents the morphometric similarity derived meso-scale cortical organisation for each participant. This was an unthresholded matrix.

For each node/ROI, we calculated both nodal degree and nodal strength. Nodal degree was the number of edges that had survived thresholding for each node. Normalised nodal strength was calculated as the ‘magnitude’ of morphometric similarity for each node. This is defined as the sum of the morphometric similarity weights of all of the edges of node *i*^70^, normalised by the degree of the node (nodes with a higher number of edges will by definition have a greater magnitude of morphometric similarity). We also calculated the average nodal strength across the network to provide a global measure of the magnitude of morphometric similarity.

In subsequent exploratory analyses we investigated the thresholded matrix across multiple network densities (threshold x = 5–40 in increments of 5), retaining only x% strongest absolute values of morphometric similarity across the graph. This has the effect of removing potential false-positive ‘edges’ of morphometric similarity. Metrics were calculated as per the unthresholded matrix.

### Sampling for training, validation and testing samples

The ABIDE cohort were divided into training, and test samples in the ratio of 6:2 (n = 245, & n = 82 respectively). Sampling for the training sample was selected pseudo-randomly, via stratified under sampling based upon age. The entire sample was binned into 0.5 yr bins dependent on age at scanning, up to the cut-off criteria of 17 years. Bins for ages 6–9 yrs were collapsed due to the much lower participant numbers in this lower tail of the distribution. Participants were equally sampled from each bin to derive the final training sample size. The training cohort was further subdivided into an internal training and validation cohort at a ratio of 5:1 (n = 204 & n = 41).

### Brainage prediction models

Brainage prediction was conducted across two, kernel-based regression approaches using the Kernlab package in R^71^; a) Gaussian Processes Regression (GPR) and b) Relevance Vector Regression (RVR). These were selected as these are both commonly used in the Brainage literature^9, 10^. Two different kernels were tested for each algorithm: a) laplacedot (Laplace radial basis kernel) and b) RBFDot (Gaussian radial basis function). Algorithm and kernel selection was conducted based upon performance on the internal validation set.

A GPR/RVR model was defined, with chronological age as the dependent variable and the morphometric data (for each of the feature sets) as the independent variables, to build a model of ‘healthy’ structural brain development. Prediction utilized 10 different feature-sets; i–vii) each of the individual morphometric features, viii) all features, ix) nodal-level strength of the morphometric similarity graph and x) edge-level weights of the morphometric similarity graph. Final model evaluation was conducted based upon performance on the independent test cohort.

In all cases, performance was evaluated based upon reducing mean absolute error (MAE) and maximizing predictive R^2^. Standard linear regression R^2^ is a biased estimate of model performance especially at lower performances^72^, whereas predicted R^2^ is more appropriate for quantifying regression accuracy^73^, calculated as;

1 $$Predicted \; {R}^{2}= 1-Normalised \; MSE$$

where the normalised MSE (Mean Squared Error) can be expanded to;

2 $$Predicted \; {R}^{2}= 1- \frac{MSE \; (Predicted \; Value,\; Observed \; Value)}{MSE \; (Observed \; Value, \; Mean \; Value)}$$

## Robustness of brainage models

### Robustness to sampling

To assess robustness of models to the sampling partitions of the data, mean and confidence intervals of predictive R^2^ values are calculated. We carried out 100 random partitions (training /testing cohort) of the data and repeated analyses to generate a vector of 100 predictive R^2^ values for the testing set from which we can take a mean metric and assess the 95% confidence interval (see Table 4).

### NHST of models

To assess the ‘real effect’ of models in comparison to ‘null’ models, we used permutation testing to conduct null hypothesis significance testing (NHST). We established the null hypothesis as no meaningful patterns in the data between age and feature sets in training, and thus poor performance on the test cohort. To derive such models, we permuted (n = 1000) the dependent variable of age in the training cohort and reran the models. These were then evaluated on the testing cohort where the true actual age was used and the ‘null’ R^2^ was calculated. The mean predictive R^2^ values of the resampled models (above), and the distribution of R^2^ values from the permuted ‘null’ cases allowed calculation of *p*-values where the frequency of instances in the distribution where the mean predictive R^2^ was greater than that of the null models. Significance of *p*-values was assessed at the level of Bonferroni corrected α < 0.005, corrected over the 10 models.

## Data Availability

The data used in this research was acquired through the public Autism Brain Imaging Data Exchange (ABIDE, Di Martino, Yan^31^) database. Specifically, we used the ABIDE data release as shared by the Preprocessed Connectome Project (PCP, Bellec, Yan^32^. For full details see Pre-processed Connectome Project website http://preprocessed-connectomes-project.org/). The R code is also available from the authors upon request, however all open-source packages used in the study are listed here: *data.table, scales, psych, ggplot2, neuroCombat, ggseg, dplyr, ggpubr, ggExtra, kernlab, ppcor, PupillometryR, tidyr*.
